# Photodynamic Inactivation of *Candida albicans* with Imidazoacridinones: Influence of Irradiance, Photosensitizer Uptake and Reactive Oxygen Species Generation

**DOI:** 10.1371/journal.pone.0129301

**Published:** 2015-06-08

**Authors:** Aleksandra Taraszkiewicz, Grzegorz Szewczyk, Tadeusz Sarna, Krzysztof P. Bielawski, Joanna Nakonieczna

**Affiliations:** 1 Laboratory of Molecular Diagnostics, Intercollegiate Faculty of Biotechnology University of Gdansk and Medical University of Gdansk, Gdansk, Poland; 2 Department of Biophysics, Faculty of Biochemistry, Biophysics and Biotechnology, Jagiellonian University, Krakow, Poland; Massachusetts General Hospital, UNITED STATES

## Abstract

The increasing applicability of antifungal treatments, the limited range of available drug classes and the emergence of drug resistance in *Candida* spp. suggest the need for new treatment options. To explore the applicability of *C*. *albicans* photoinactivation, we examined nine structurally different imidazoacridinone derivatives as photosensitizing agents. The most effective derivatives showed a >10^4^-fold reduction of viable cell numbers. The fungicidal action of the three most active compounds was compared at different radiant powers(3.5 to 63 mW/cm^2^), and this analysis indicated that 7 mW/cm^2^ was the most efficient. The intracellular accumulation of these compounds in fungal cells correlated with the fungicidal activity of all 9 derivatives. The lack of effect of verapamil, an inhibitor targeting *Candida* ABC efflux pumps, suggests that these imidazoacridinones are not substrates for ABC transporters. Thus, unlike azoles, a major class of antifungals used against *Candida*, ABC transporter-mediated resistance is unlikely. Electron paramagnetic resonance (EPR)-spin trapping data suggested that the fungicidal light-induced action of these derivatives might depend on the production of superoxide anion. The highest generation rate of superoxide anion was observed for 1330H, 1610H, and 1611. Singlet oxygen production was also detected upon the irradiation of imidazoacridinone derivatives with UV laser light, with a low to moderate yield, depending on the type of compound. Thus, imidazoacridinone derivatives examined in the present study might act via mixed type I/type II photodynamic mechanism. The presented data indicate lack of direct correlation between the structures of studied imidazoacridinones, cell killing ability, and ROS production. However, we showed for the first time that for imidazoacridinones not only intracellular accumulation is necessary prerequisite of lethal photosensitization of *C*. *albicans*, but also localization within particular cellular structures. Our findings present IA derivatives as efficient antifungal photosensitizers with a potential to be used in local treatment of *Candida* infection.

## Introduction

Due to longer life span and increasing numbers of people with compromised immune systems, the number of fungal infections caused by yeasts has significantly increased in recent years[1]. This phenomenon is also related to a dramatic increase in antifungal drug resistance. The newest classes of antifungals (e.g., triazoles and echinocandins) are widely used and effective, however, optimal therapy may be complicated through emergence of at least four resistance mechanisms, the most prominent being the induction of efflux pumps leading to low drug concentration [2]. Antimicrobial agents that can be activated with light are commonly known as photoantimicrobials [3]. In the presence of oxygen, these agents use the energy of visible light to produce highly reactive oxygen species, e.g., singlet oxygen or hydroxyl radicals, which are toxic to microbial cells. The process is known as antimicrobial photodynamic inactivation (PDI). The main advantage of photoantimicrobial agents, also known as photosensitizers, over classical antimicrobials is the multiple-target actions of these compounds. Reactive oxygen species, as long as they are localized in the close vicinity of a specific molecular target, act on proteins, nucleic acids or unsaturated lipids. As a consequence, photoantimicrobials are relatively immune to individual resistance mechanisms, and can act effectively against wild-type and antifungal-resistant isolates. Indeed, there has been no reports indicating the *in vitro* induction of resistance to the action of light-dependent drugs [4–6]. Due to their short life times, reactive oxygen species act locally. The selectivity issue remains a concern, particularly in the case of yeast cells (i.e., eukaryotic pathogens), but the potential ‘therapeutic window’ although more narrow compared with bacterial cells, is still possible to obtain [7–8]. Photoantimicrobials are only active after light treatment, thus localized skin and soft tissue infections caused by fungi are good targets for PDI. Thus, this treatment assures the selectivity and minimization of the side effects.

The fluence of light has been, for years, considered the most important parameter in photodynamic action against microbial cells, based on the reciprocity principle [9]. However, some well documented reports exist pointing to the importance of the time parameter over which the light is being delivered to cells [10]. Influence of two fluence rates of light (artificial or solar) on the efficiency of photoinactivation was studied in the past with the use of cationic porphyrins, where it was demonstrated that under 620 W/m^2^ of solar light, photoinactivation of bioluminescent *E*. *coli* was faster over artificial 40 W/m^2^ due to higher light fluence rate [11]. The effect of light source, light dose, fluence rate was also shown to be of critical meaning for phage photoinactivation in a extensive studies using light sources within range of 40 W/m^2^–1690 W/m^2^[12].The finding that fluence rate and exposure time play important role in effectiveness of antimicrobial PDI is of profound meaning and opens the possibility to optimize irradiation protocols for *in vivo* studies.

*Candida* spp. plays a major role in localized and systemic infections in humans. The fungal infections are particularly risky for a growing number of immune-compromised, and post-trauma patients, individuals with tumours and those who underwent surgical interventions. *Candida albicans* is most frequently isolated fungi species from infections [13].

Several studies on planktonic and biofilm forms of *C*. *albicans* culture have shown effective killing using various photosensitizing compounds, including phenothiazinium photosensitizers [7,14–16], phthalocyanines[17] and porphyrins[18–24]. Antifungal PDI research is currently at the stage of *ex vivo* experiments, i.e., using yeast cell cultures, rather than infected animal models. However, some *in vivo* studies on animal models of human infections demonstrated the potential of PDI in eradicating pathogenic fungi. Indeed, a significant decrease in the number of viable *C*. *albicans* cells was observed following PDI in rat model of oral candidiasis using a hematoporphyrin derivative[25] or methylene blue[26].However, the complete elimination of viable cells was not achieved. In other studies, antifungal PDI was shown to reduce microscopic lesions of experimentally induced candidiasis in rats and inhibit the proteinase activity of *C*. *albicans*. Albeit, a reduction in the number of viable cells was not achieved[27,28]. The adequate efficacy of a new methylene blue-based PDI was also established for a cutaneous skin infection[29] in rat model of skin burn wound infections[30], and in *C*. *albicans*-induced murine vaginitis[31]. Thus, despite considerable progress, antifungal PDI remains at the stage of development, and in particular, the effective killing of pathogen cells remains a challenge. Therefore, there is a need for the development of new and improved photoantimicrobials, and the examination of the mechanism underlying actions of these compounds.

The objective of the present study were therefore:(i) to investigate the irradiance-dependent efficacy of *C*. *albicans* photoinactivation with the use of imidazoacridinone derivatives, (ii) to study cellular accumulation of these derivatives, and the type of reactive oxygen species produced during the photodynamic process.

## Materials and Methods

### Strains

*Candida albicans* Quality Control strains were used: ATTC 10231, ATCC 90028, and ATCC 14053. For photoinactivation studies and imidazoacridinone accumulation experiments, the yeast cells were grown overnight in BHI medium (Brain Heart Infusion) with shaking (37°C, 150 rpm). ATCC strain 10231 was used in photoinactivation experiments (impact of variable irradiant power under a fixed light dose), and examining imidazoacridinone accumulation, and apoptosis induction. The latter two strains, ATCC 90028 and ATCC 14053were used in accumulation studies, and we also assessed the reduction of survival for these two strains under particular illumination conditions: 20J/cm^2^, 7mW/cm^2^).

### Chemicals

**Fig 1:**
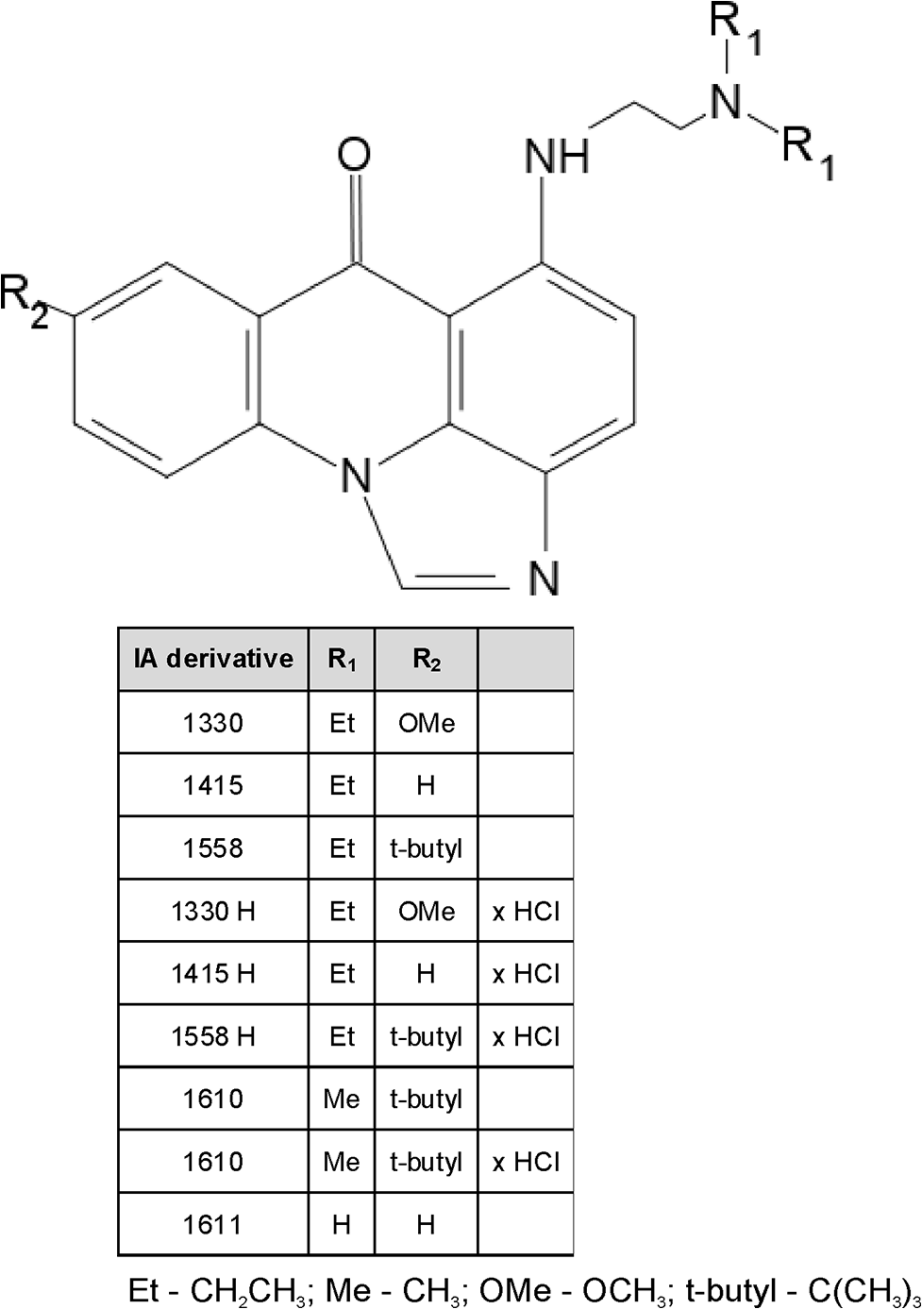
Chemical structures of imidazoacridinone derivatives.

The basic forms of three imidazoacridinone derivatives (IA), namely 1330, 1415, and 1558 were obtained from A. Skladanowski of Gdansk University of Technology, Poland. These compounds were prepared according to the method described previously [32–36]. Compounds 1610 and 1611 were synthesized according the same method as IA described above [32–36]. The hydrochloride form of the following imidazoacridinone derivatives, namely 1330H, 1415H, 1558H, and 1610H were prepared using the same protocol, and subsequently converted into hydrochloride salts using the following procedure: a slight molar excess of hydrogen chloride in absolute ethyl ether was added dropwise to the compound solution in the mixture of chloroform:methanol, at temperature 5°C. The yellow-orange solid was precipitated using anhydrous ethyl ether, followed by separation and crystalization with the ethyl ether. The general structure of the compounds used in the study is presented in Fig 1. The compounds were first dissolved in pure (100%) DMSO (Sigma-Aldrich, Germany) to obtain 10 mM stock solutions, and stored at -20°C until further used.All other chemicals were of analytical grade, including meso-Tetra(N-methyl-4-pyridyl)porphinetetratiosylate (TMPyP) (Sigma-Aldrich, Germany); Verapamil (Sigma-Aldrich, Germany); 5,5-Dimethyl-1-pyroline *N*-oxide (DMPO)(Sigma-Aldrich, Germany).

### Antimicrobial photodynamic studies on *C*. *albicans*

*Candida albicans* was grown overnight in standard BHI (Brain Heart Infusion) medium (Biomerieux, France) at 37°C, with shaking (150 rpm). *C*. *albicans* standardized suspension in BHI (5x10^6^ CFU/ml) was prepared and added to the wells on sterile 96 well flat-bottom microtiterplate. The studied concentrations (50 or 100 μM) of each photosensitizer imidazoacridinone derivative were added. The plate was incubated in the dark at 37°C for 30 min without shaking and the samples were irradiated (LED illuminator, output power of 11W/12mm^2^, blue light of 405 nm, custom designed for laboratory use(Secure Media, Poland). After irradiation serial dilutions were obtained from every sample in sterile PBS and 10 μl aliquots were spotted on BHI agar plates. The plates were incubated overnight at 37°C and the colony forming units (CFU) were counted and the results were statistically analysed. Three types of controls were included in the photoinactivation experiment: (i) cells treated with light only, (ii) cells incubated in the dark in the presence of imidazoacridinone derivatives, and (iii) cells incubated in the dark in the absence of imidazoacridinones. Each experiment was performed as a separate biological replicate from cultures grown independently.

### Accumulation of photosensitizers in *C*. *albicans*

Standardized suspensionsof *C*. *albicans* were prepared in PBS (10^6^ CFU/ml) from overnight grown independent cultures in BHI (Brain Heart Infusion) medium (Biomerieux, France). Each assay was performed as a separate biological replicate of the experiment. Photosensitizers (IAs) were added at concentrations of 50 and 100 μMto 1 ml of *C*. *albicans* suspension. The samples were incubated in the dark at 37°C for the 30 min without shaking. Samples were centrifuged at room temperature for 5 mins, at 5000 rpm (2.3xg), and supernatant was spectrophotometrically analysed to determine the amount of photosensitizer remaining outside the cells. The imidazoacridinone concentrations were calculated based on the Lambert-Beer law and the extinction coefficient values were experimentally measured for each compound. The concentrations of accumulated imidazoacridinone in these cells were obtained after subtracting the calculated concentration of imidazoacridinone present in the supernatant from the concentration used for cell incubation (50 or 100 μM). These values are presented in Table 1 (IAs accumulated in cells). Subsequently, the cells were centrifuged at room temp for 5 mins, at 5000 rpm (2.3xg), re-suspended in PBS, and further incubated in the dark at 37°C for 30 min without shaking. The cells were subsequently pelleted (5 min, 5000 rpm [2.3xg], room temp.),and absorbance of the supernatants was analysed to determine the amount of the photosensitizers transported out of the cells (Table 1, IAs remained after washing). The absorption measurements were performed on the Beckman DU-640 Spectrophotometer at 420 nm. To measure imidazoacridinones accumulation in the presence of verapamil, the cells (5 McFarland, 10^6^CFU/ml) were centrifuged at room temp for 5 mins, at 5000 rpm (2.3xg), washed with PBS, incubated with 200 μM verapamil, and subsequently, without further washing, incubated with each imidazoacridinone derivative. the cells were then pelleted under the above conditions and the absorbance of the supernatants was measured.

**Table 1:** Accumulation and phototoxic effect of imidazoacridinones in *Candida albicans* ATCC 10231 strain.

IAs	Initial IAs conc. 50 μM = 0.05 μmol/10^6^ cells		Initial IAs conc. 100 μM = 0.1 μmol/10^6^ cells		PDI effect (20 J/cm^2^, 7 mW/ cm^2^)	
	IAs accumulated in cells^a^	IAs remained after washing^a^	IAs accumulated in cells^a^	IAs remained after washing^a^	50 μM IA	100 μM IA
	μmol/10^6^ cells ± SD		μmol/10^6^ cells ± SD		Reduction in survival [log_10_ units CFU/ml]^b^ ^,^ ^c^ ^,^ ^d^	
**1330**	0.033 ± 0.002	0.023 ± 0.002	0.076 ± 0.004	0.064 ± 0.0067	2 ± 0.03	>5 ± 0.00
**1415**	0.023 ± 0.002	0.013 ± 0.002	0.054 ± 0.006	0.036 ± 0.0066	1 ± 0.1	1 ± 0.1
**1558**	0.031 ± 0.001	0.020 ± 0.005	0.065 ± 0.010	0.048 ± 0.0119	0 ± 0.5	2 ± 0.1
**1330h**	0.042 ± 0.001	0.032 ± 0.007	0.086 ± 0.001	0.077 ± 0.0048	1 ± 0.22	2 ± 0.19
**1415h**	0.019 ± 0.002	0.006 ± 0.002	0.042 ± 0.002	0.024 ± 0.0036	1 ± 0.13	1.5 ± 0.22
**1558h**	0.024 ± 0.002	0.010 ± 0.004	0.049 ±0.004	0.026 ± 0.0065	0 ± 0.65	1 ± 0.12
**1610**	0.036 ± 0.002	0.023 ± 0.003	0.065 ± 0.004	0.046 ± 0.0055	1 ± 0.00	2.5 ± 0.00
**1610h**	0.035 ± 0.004	0.026 ± 0.011	0.063 ± 0.003	0.044 ± 0.0062	1 ± 0.55	2 ± 0.11
**1611**	0.011 ± 0.002	0.013 ± 0.010	0.028 ± 0.004	0.022 ± 0.0047	0 ± 0.80	0 ± 0.07

IAs—imidazoacridinone derivatives; PDI—antifungal photodynamic effect, The values were calculated by subtracting log_10_ CFU/ml of tested samples from those of untreated controls (0 J/cm^2^; 0 μM IA). At least three biological replicates were used for calculation of the mean reduction values.

^a^mean values of three replicates ± standard deviation of the mean

^b^in samples incubated in the dark without imidazoacridinone derivatives, the number of *C*. *albicans* cells was c.a. 5x10^6^ CFU/ml

^c^in the samples incubated in the dark with 50 μM or 100 μM of each imidazoacridinone derivative, the number of cells was c.a. 5x10^6^ CFU/ml

^d^in the samples exposed to light only (20J/cm^2^, 7mW/cm^2^), the number of *C*. *albicans* cells was c.a. 5x10^6^ CFU/ml

Imidazoaridinone derivatives fluorescence was observed using fluorescent microscopy. The cells (10^6^ CFU/mL) were harvested at room temp for 5 mins, at 5000 rpm (2.3xg), washed with PBS, and incubated with 50 μM of imidazoacridinone suspension. Subsequently, the cells were placed ontoa cohesive microscopic slide, covered with a glass coverslip and observed under fluorescent microscopy at an excitation of 360–370 nm, and emission of >420 nm (Olympus BX51 fluorescence microscope with F-View-II CCD camera). The measurements and image analysis were conducted using AnalySIS software.

### Time-resolved spectroscopic detection of singlet oxygen -^1^O_2_

Time-resolved luminescence of ^1^O_2_ was measured at 1270 nm. Solutions of imidazoacridinones (100 μM) in 1-cm fluorescence cuvettes (QA-1000, HelmaOptik) were excited with 355 nm microjoule pulses (750 ps duration) generated using a microchip Nd:YAG laser (Pulselas-P-1064-FC, Alphalas GmbH, Germany) operating with a 2–10 kHz repetition rate. To filter out the first and third harmonics of laser radiation, 50-cm water filter and dichroic mirrors (BK7series, Eksma Optics, Lithuania) were used. Near-infrared luminescence was measured perpendicularly to the excitation beam in a photon counting mode using a thermoelectric cooled NIR PMT module (Model H10330-45, Hamamatsu, Japan) equipped with 1100-nm cutoff filter and additional selected narrow-band filters (NB series, NDC Infrared Engineering LTD, UK). A computer-mounted PCI-board multichannel scaler was used (NanoHarp 250, PicoQuant GmbH, Germany), and the data collection was synchronized with laser pulses using an ultrafast photodiode (UGP-300-SP, Alphalas GmbH, Germany) as a trigger. First-order luminescence decay fitting was achieved using the Levenberg-Marquardt algorithm in custom-written software. Each measurement of singlet oxygen phosphorescence was repeated three times on independent samples.

### Detection of oxygen radicals through Electron Paramagnetic Resonanse spin trapping

EPR spin-trapping was employed using DMPO as a spin trap at concentrations of 10 mM. Samples of imidazoacridinone derivatives (100 μM each) were irradiated in EPR quartz flat cells in the resonant cavity with blue light (70 mW/cm^2^) derived from a 300 W high pressure compact arc xenon lamp (Cermax, PE300CE–13FM/Module300W, Perkin-Elmer) equipped with a water filter, heat reflecting hot mirror, cutoff filter blocking light below 390 nm, and dichroic filter transmitting light at a range of 400–500 nm. The EPR samples were run using a microwavepower of 10.55 mW, modulation amplitude of 0.05 mT, scan width 8 mT, and a scan time of 80 s, while the kinetics measurements were obtained at an overmodulated amplitude (0.2 mT).The EPR measurements were performed using a BrukerEMX–AA EPR spectrometer (BrukerBioSpin, Germany). Simulations of EPR spectra were performed usingWinSIM (Version 0.98) software. TheEPR spin trapping measurements were carried out using three independent samples yielding with very similar results.

### Statistical analysis

Each experiment was performed at least in triplicate. The primary data are presented as the means with standard deviations of the mean. The statistical analysis was performed using one-way analysis of variance (ANOVA) with Tukey’s post-hoc test. The hypotheses were tested at a significance level of 0.05. All analyses were performed using the STATISTICA version 8.0 software (StatSoft Inc. 2008, data analysis software system, Tulsa, USA).

## Results

### Irradiance influences the antifungal PDI outcome

**Fig 2:**
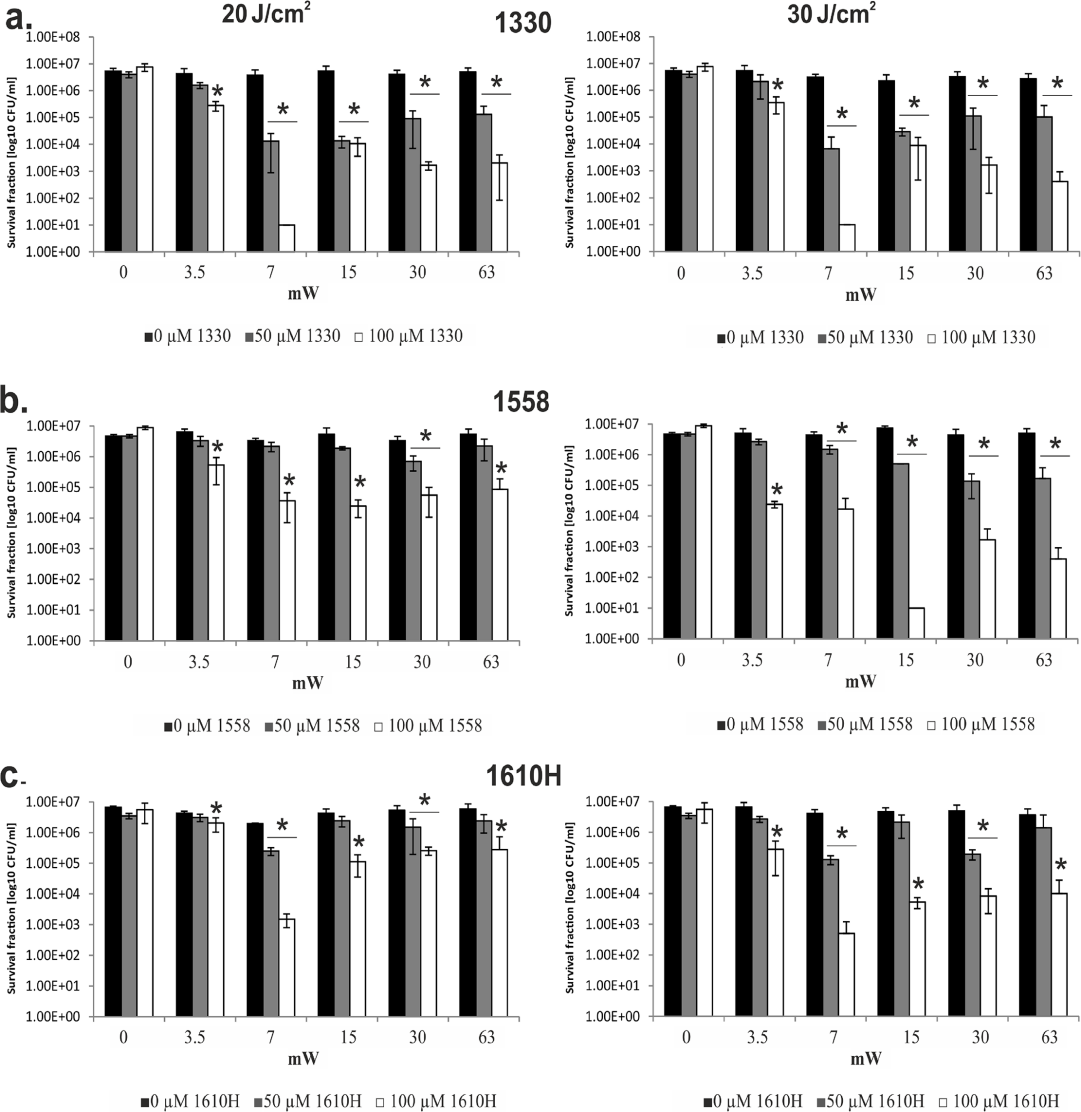
Survival of *Candida albicans* ATCC 10231 cells after photodynamic treatment with various irradiance power and imidazoacridinone derivatives. Three representative imidazoacridinone derivatives are shown here: **a.** 1330, **b.** 1558, and **c.** 1610H. The cells were incubated with the following concentrations of each imidazoacridinone derivative: 0, 50, and 100 μM. Following incubation, a total light dose (LED light 405 nm) of 20 J/cm^2^ (**left panel**) or 30 J/cm^2^ (**right panel**) was applied, at various irradiance power as indicated on the X-axis. The value of ‘0 mW’ represents control experiments, i.e., cells incubated in the dark in the absence (0 μM) or presence (50 μM or 100 μM) of IA derivative, and not subjected to illumination. The remaining values of ‘3.5 mW’ up to ‘63 mW’ represent the light-treated samples at a given irradiance. The statistical significance of the reduction in survival was calculated for each IA-treated sample with respect to the light only treated samples (black bars). The number of surviving cells presented on the Y-axis represents the mean value of three independent biological replicates, and the error bars represent the standard deviation. *, significant at the level of P value of < 0.05.

We first examined the impact of variable irradiant power under a fixed light dose. Hence, the time of irradiation was accordingly varied, testing 3 different most active imidazoacridinone derivatives, 1330, 1558 and 1610H and measuring the survival of yeasts cells. The total dose of light applied was 20 J/cm^2^, and the dose rate varied at 3.5, 7, 15, 30, and 63 mW/cm^2^. The results indicated a non-linear correlation between the applied power density and cell killing. Neither the highest applied power density (of 63 mW/cm^2^, the shortest exposure time), nor the lowest power density(3.5mW/cm^2^, the longest irradiation time)were effective. The best results were obtained at medium irradiance (7 mW/cm^2^) and hence, at a medium irradiation time (Fig 2). A similar pattern of medium irradiance being most active in photokilling, was observed at a higher light dose (30 J/cm^2^; Fig 2). However, a dose rate of7 mW/cm^2^ was the most effective in 1330 and 1610H activation, whereas 15 mW/cm^2^ was the most efficient in case of 1558 activation. The compound 1330 was previously photoactivated at a lower light dose of 20 J/cm^2^, so that further increase to 30 J/cm^2^ had no significant effect on photokilling. However, for the remaining 2 compounds, 1558 and 1610H, the higher light dose applied exerted improved killing efficacy, reaching 5 log_10_ units of killing at 30 J/cm^2^ versus only 2 log_10_ units at 20 J/cm^2^ as observed for 1558 (15 mW/cm^2^). A similar tendency was observed for 1610H, although the differences at 7 mW/cm^2^ between optimal photokilling with a total light dose of 30 J/cm^2^ versus 20 J/cm^2^ were not so explicitly pronounced: 3.5 log_10_ units of killing and 3 log_10_ units of killing, respectively (Fig 2). Overall, we observed that, beyond the light dose itself, the time of exposure, and hence the irradiation power is a critical determinant of the effectiveness of antimicrobial photoinactivation.

### Imidazoacridinones accumulation in *Candida albicans*

We examined 3 ATCC reference strains of *C*. *albicans* (10231, 90028, and 14053) to quantify the amounts of photosensitizers that accumulated within these cells. We applied the spectrophotometric measurements, as described in Materials and Methods. We observed no significant differences in the accumulation profiles among the studied strains with respect to the examined imidazoacridinone derivative (Table 1, S1–S2 Tables). Therefore the next analysis focused on *C*. *albicans* strain 10231.

The quantity of imidazoacridinones accumulated in *C*. *albicans* strain 10231 after incubation with 50 μM (0.050 nmol/10^6^ cells) of the tested compounds ranged from 0.015 to 0.042 nmol/10^6^ cells (Table 1). Compound 1611 was an exception, exhibiting the lowest value of accumulation (0.01 nmol/10^6^ cells). This low accumulation level correlated with a lack of the phototoxic activity at both 50 and 100 μM concentrations tested (Table 1). The compounds, resulting in the highest photokilling, namely 1330, 1330H, 1558, 1610 and 1610H, accumulated most effectively (more than 60% of initial concentration used). Moreover, for the most photoactive compounds, the amount remaining within the cells after washing with PBS, was > 70% of the initially accumulated amount. Notably, 1611, which did not exhibit any phototoxic activity, accumulated in *C*. *albicans* cells only at 20%, and this level remained unchanged after PBS washing.

**Fig 3:**
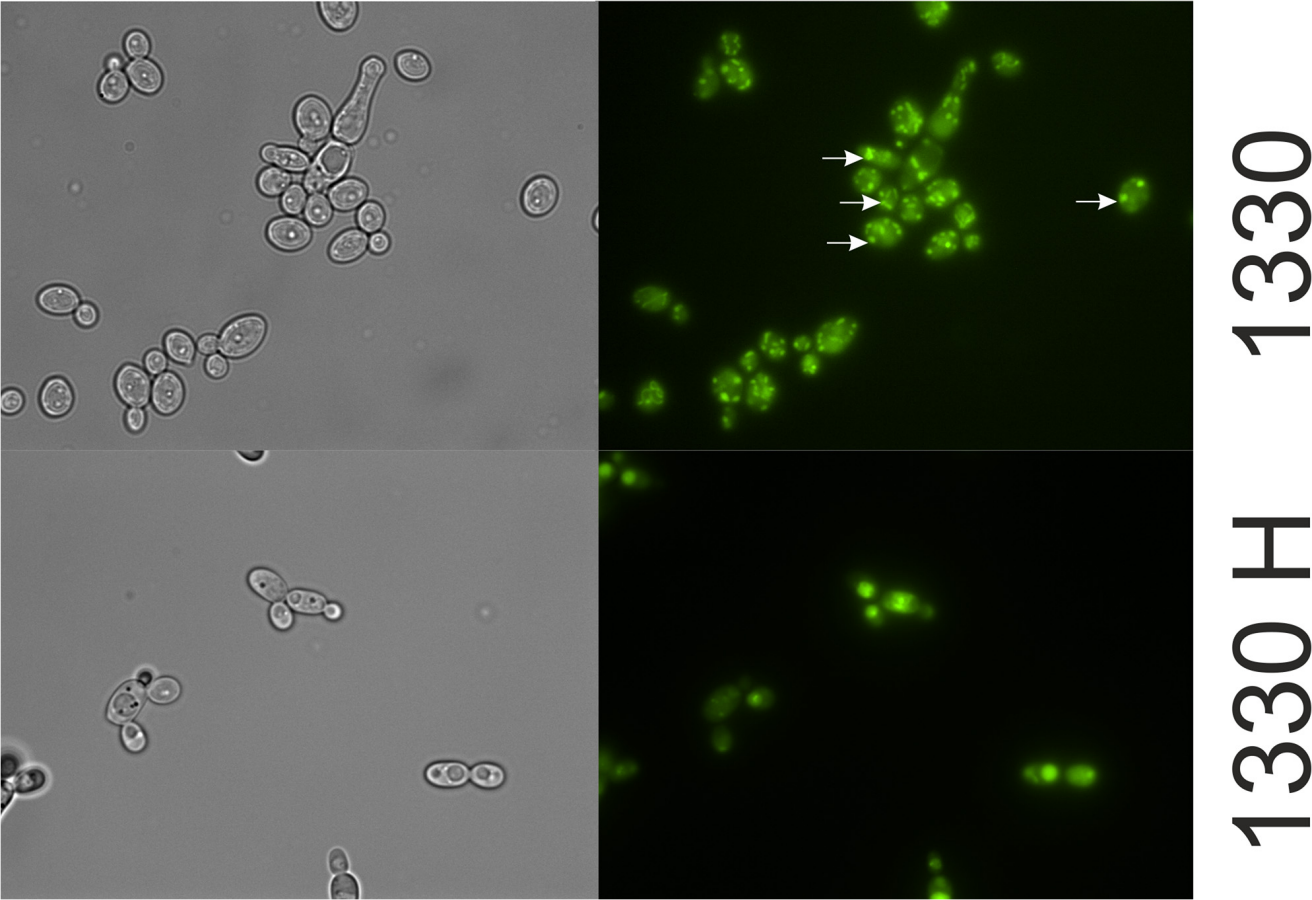
Accumulation of imidazoacridinone derivatives in *Candida albicans* ATCC 10231 cells. The cells were incubated in the dark with the denoted imidazoacridinone derivatives (50 μM, 30 min, 37°C). Imidazoacridinone fluorescence (right panel) was observed under a fluorescence microscope (excitation wavelength 360–370 nm, emission >420 nm). The same cells visualized under white light are presented in the left panel.

We next examined the accumulation of the applied imidazoacridinone derivatives in *C*. *albicans* strain 10231 using fluorescent microscopy. The optimal imidazoacridinones concentration and time of incubation in the dark were selected based on previous studies [37]. The fluorescent microscope images (Fig 3), revealed quantitative differences in accumulation and the distribution pattern inside the cells. For example, after 30 min of incubation, 1330H was observed inside the cell, with the highest concentration detected within the subcellular membrane fraction. This trend was similar with 1558H, 1610, and 1610H. The remaining two derivatives, namely 1611 and 1415H, accumulated poorly compared with the four above mentioned compounds, and showed a more peripheral distribution. A completely different pattern of intracellular distribution was observed for 1330. In this case, the signal corresponded with small, explicit fluorescing points distributed inside the cell (Fig 3).

### Verapamil inhibits imidazoacridinone accumulation

**Fig 4:**
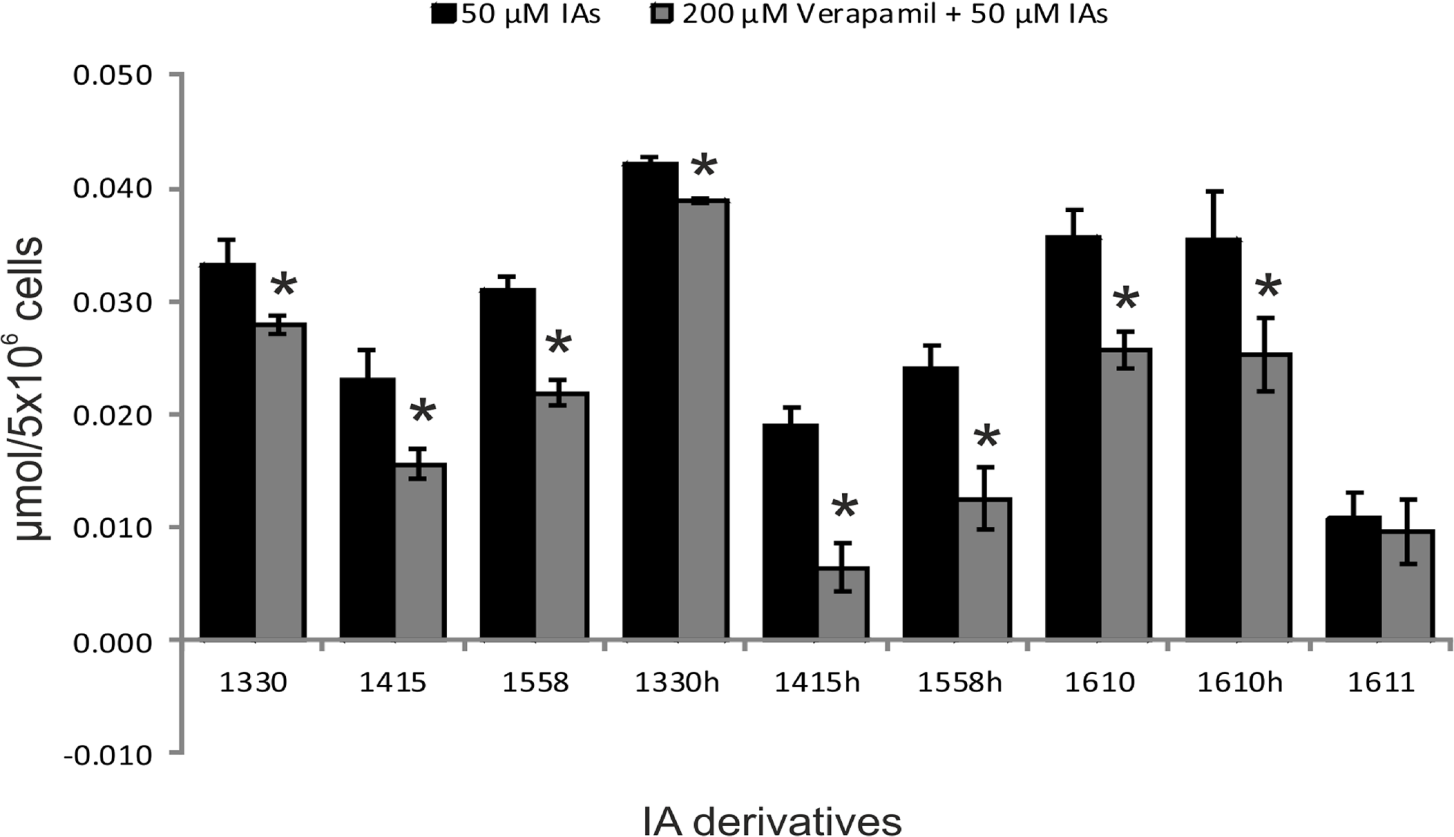
Accumulation of imidazoacridinone derivatives in *Candida albicans* ATCC 10231 cells in the presence of verapamil. The cells (10^6^ CFU/ml) were incubated with 50 μM of a particular imidazoacridinone derivative (indicated on the X-axis) in the dark, and the accumulation was measured through spectrophotometry as described in Materials and Methods (**black bars**). The cells treated as described, but pretreated with 200 μM of verapamil before incubation with the imidazoacridinone derivative, are represented with **grey bars**. The mean values from three independent biological experiments are shown and the error bars represent the standard deviation.*, significant at the level of P value of < 0.05.

We determined whether the ABC transporters in *C*. *albicans* recognized the newly applied IAs, and whether resistance to PDI based on these photosensitizers might occur. To this end, we pre-treated *C*. *albicans* with verapamil (200μM), a potent ABC pump inhibitor, and subsequently measured the levels of IA accumulation. The results, presented in Fig 4, indicated a decrease in IAs accumulation after verapamil pre-treatment, consistent with free diffusion rather than active transport. We also observed differences between the overall accumulation levels and accumulation in the presence of verapamil with respect to particular compounds. Verapamil had negligible effect on the most effectively accumulated compounds (1330 and 1330H; the estimated decrease in accumulation in the presence of verapamil accounted for 15% and 8%, respectively compared with the amount accumulated in the absence of verapamil). In case of 1415, 1558, 1610, and 1610H verapamil pre-treatment had a more noticeable effect (30% decrease in accumulation with respect to non-verapamil treated samples). The highest decrease in accumulation after verapamil treatment was observed for 1415H and 1558H (> 60%). The effect was apparent for 1611, as yeast cells accumulated a low amount of this compound and verapamil had no visible effect on this process (Fig 4).

### Photochemistry of imidazoacridinones in model systems

We used two experimental approaches to determine the type of photochemical processes mediated through imidazoacridinones in simple model systems. The approaches included: near-infrared time-resolved luminescence, a direct method to monitor the formation and decay of singlet oxygen (^1^O_2_), and EPR-spin trapping, an indirect method for detecting short-lived free radicals. It is important to emphasize that EPR-spin trapping is a canonical method for studying oxygen radicals formed during type I photosensitized oxidation reactions.

**Fig 5:**
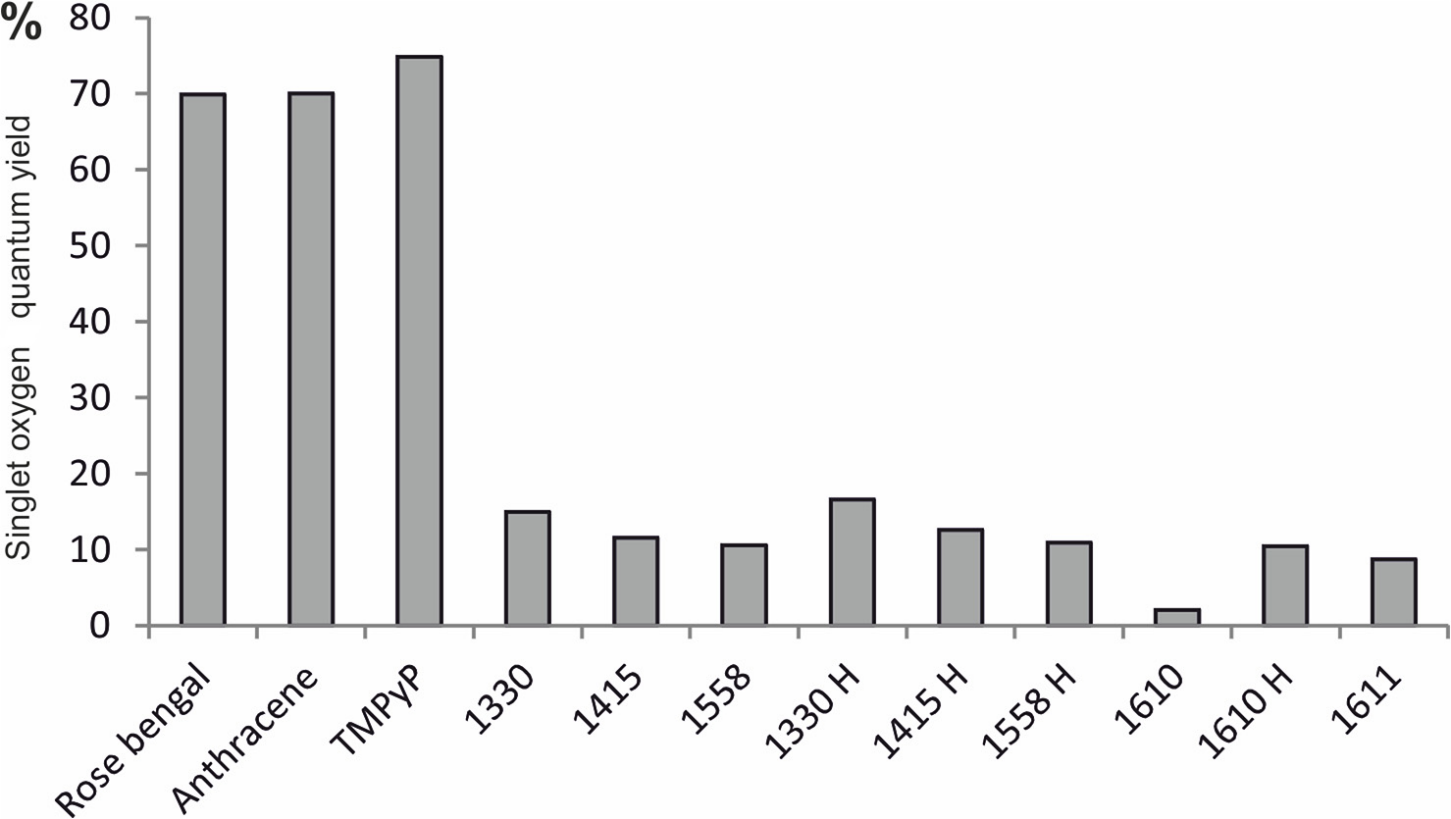
Singlet oxygen generation through imidazoacridinone derivatives. Time-resolved luminescence of singlet oxygen (^1^O_2_) was measured at 1270 nm. The excitation wavelength used was 355 nm. Rose Bengal, anthracene, and TMPyP were used as standards with known singlet oxygen quantum yields. The Y-axis represents the quantum yield of singlet oxygen generation, where a maximal value of 100% indicates that the entire energy of triplet state is transformed into a singlet excited state of molecular oxygen. The quantum yields are determined within +/- 5% accuracy.

**Fig 6:**
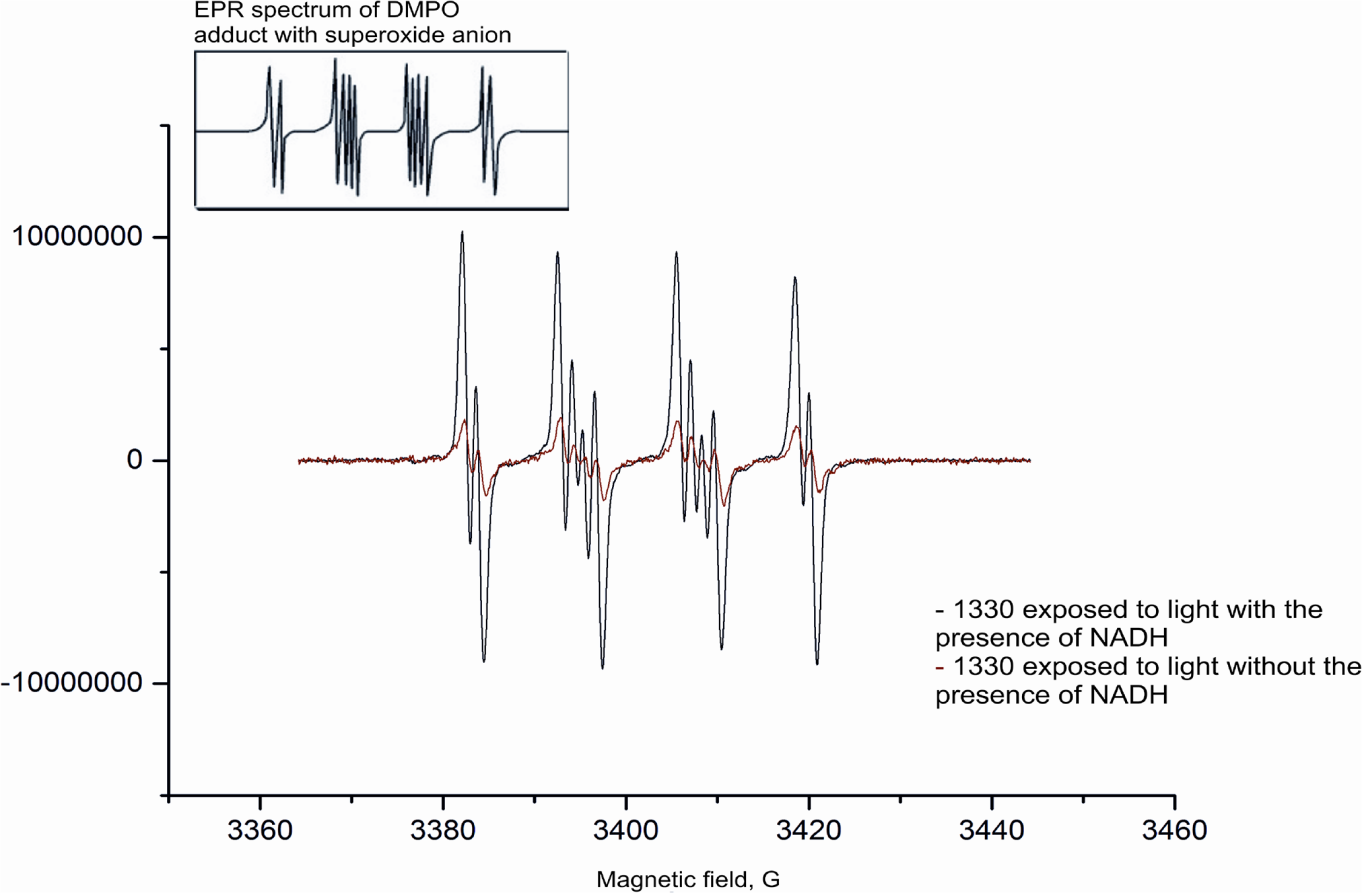
A representative single Electron Paramagnetic Resonance (EPR) spectrum obtained upon photoexcitation of imidazoacridione derivative 1330. The predicted (inlet) and experimental spectra of photoexcited imidazoacridinone 1330 in a DMSO/DMPO mixture, upon exposure to light at a wavelength range of 540–740 nm are shown.

**Fig 7:**
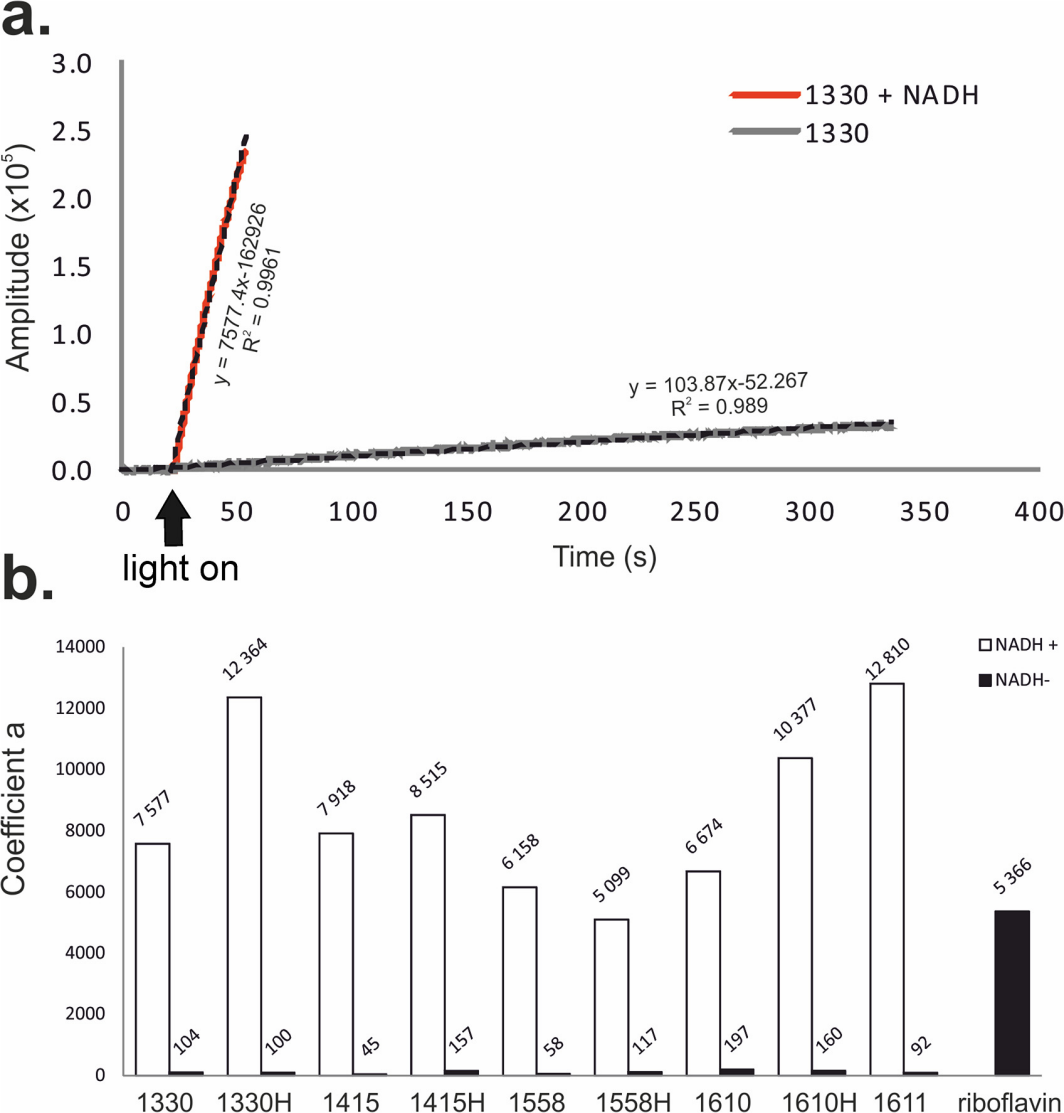
Electron Paramagnetic Resonance (EPR)-spin trapping experiments. **a.** An example of an EPR spectrum of superoxide anion created upon photoexcitation of imidazoacridinone 1330 alone and in the presence of NADH. **b.** Comparison of the *coefficient a* indicating the efficiency in superoxide anion creation derived from the EPR spectra of different irradiated imidazoacridinone derivatives (indicated in X-axis) in the presence and absence of NADH. Riboflavin was used as a positive control photosensitizer that efficiently generates superoxide anion in the absence of NADH.

Quantum yields for singlet oxygen photogeneration through IAs were determined by measuring time-resolved luminescence in acetonitrile solutions at 1270 nm, after excitation of the studied compounds with 355 nm laser light. Rose Bengal was used as a reference, following excitation at 355 nm. The photoexcitation of IAs resulted in singlet oxygen generation (Fig 5), evident by the observed dependence of the intensities of the time-resolved luminescence signals on oxygen presence in the samples studied and wavelength at which the signals were detected. Importantly, the corresponding quantum yields varied from low (2%) for 1610 to moderate (16%) for 1330H compared with the well known efficient singlet oxygen generators Rose Bengal (70%) and TMPyP (75%). Electron paramagnetic resonance (EPR)-spin trapping using 5,5-dimethyl-1-pyrroline N-oxide (DMPO) as a spin trap, revealed the photogeneration of superoxide anions (O_2_ˉ˙) through all imidazoacridinones (using as solvent DMSO:H_2_O, at 9:1 ratio). Fig 6 shows the exemplary spectra of the DMPO spin adduct after the photoexcitation of 1330 compound in the presence and absence of reduced nicotinamide adenine dinucleotide (NADH). The simulated spectrum of the DMPO spin adduct using superoxide anion is shown in the inset of Fig 6. Comparison of the simulated and experimental spectra indicates that 1330 produces superoxide anion upon excitation with UV light (355 nm) and the photoformation of the O_2_ˉ˙signal is enhanced by about seventy-fold in the presence of an electron donor such as NADH. Similar spectra were recorded for all the remaining imidazoacridinone derivatives. The accumulation of the DMPO spin adduct as a function of irradiation time for derivative 1330, in the presence and in the absence of NADH, is shown in Fig 7A. These data indicate that the relative rates of spin adduct formation can be calculated with respect to riboflavin (a reference superoxide anion generator). Such measurements performed for different imidazoacridinones indicate that the rate of superoxide anion photoformation is 30–60 times lower than that of riboflavin (Fig 7B). Notably, one of the highest rates of superoxide radical generation was observed with 1611.

Notably, the rate of O_2_ˉ˙ generation was dramatically increased for all tested imidazoacridinones when NADH was included in the reaction mixture. The observed acceleration of O_2_ˉ˙ generation in the presence of NADH, varied among the studied compounds. The highest enhancement of the rate of superoxide anion photoformation (~140-fold) was observed for 1330H, 1610H, and 1611, while the lowest rate was detected for 1610(~30-fold).

## Discussion

The apparent increase in a number of studies on *C*. *albicans* reflects two phenomena: the growing number of isolates resistant to the currently applied antifungals, and, the increasing number of patients with local candidiasis symptoms alongside the growing number of neutropenic, immune-compromised patients [38]. Thus, antifungal photodynamic therapy can be considered as an alternative promising strategy to control localized *C*. *albicans* infections [18,39]. Notably, although encouraging data on photoinactivation of *Candida* spp. have been published, there are only few reports concerning the characterization and/or mechanism of action of new photosensitizers. Here, we addressed photosensitizer cellular accumulation, the nature of generated reactive oxygen species, and light-induced photosensitizers activation. These aspects of photodynamic therapy are important, particularly regarding the potential clinical applications of antifungal PDI.

Porphyrins and phenothaziniums were the first compounds shown to be effective in the photosensitization of *C*. *albicans* cells in suspension [7,20]. These results were later confirmed in mouse models of local *Candida* infections [25,26] observing antifungal effects, including strains that were azole-resistant[40]. However, due to the variability in experimental parameters, the comparison of outcomes from different studies is difficult. When analysing photochemical and photobiological phenomena, fluence (the energy delivered per unit area) is a key radiation parameter to quantitatively characterize the observable effects. According to the Bunsen-Roscoe law (the reciprocity rule), the observable effect is directly proportional to the total radiation energy dose, irrespective of the administered regime[9]. However, it has been demonstrated that certain photobiological reactions critically depend on the fluence rate (power of radiation per unit area incident on a surface)[9]. Accordingly, Henderson *et al*. showed that low fluence rate significantly decreases the number of clonogenic cells, while an insignificant reduction was observed following the high fluence rate treatment of tumour cells with Photofrin [41]. A similar trend was also observed in the present study (Fig 2). The efficacy of photodynamic therapy could therefore be significantly increased through modifications in the irradiation protocol. The fact that fluence rates affect imidazoacridinone-mediated photodynamic treatment, may be considered as a valuable means of increasing the selectivity of phototherapy. Recent data, and the results published by another group using methylene blue [10] indicate that output power and/or time of exposure should be considered as important determinants of the photokilling of yeast cells. Similar conclusions can be drawn based on sewage bacteriophage inactivation with cationic porphyrins [12]. Although different molecular and cellular mechanisms could be responsible for the observed effects, oxygen depletion induced through photosensitized reactions, might play an important role. It is conceivable that oxygen photouptake at higher fluence rates exceeds oxygen replenishment due to its diffusion from oxygen rich surroundings. As a result, the efficiency of photodynamic cell killing will decrease at higher fluence rates.

In our previous work on 1330, we did initial experiments concerning photo- and cytotoxicity of this compound towards human HaCaT cells. We observed that 200J/cm^2^ of 380–480 nm light only treatment causes about 25% decrease in the survival of human HaCaT cell line with respect to non-light treated cells [37]. This indicates certain degree of light only toxicity, however, still at an acceptable level. With the following PDI treatment conditions: 200J/cm^2^, 50 μM of 1330 compound, we obtained satisfactory therapeutic window (3.1 log_10_ decrease in *C*. *albicans* cells survival, and 43% decrease in HaCaT cell line survival), which points out that C1330 may potentially be used as a candidate photosensitizer in the light-dependent killing of *C*. *albicans* cells.

Another key factor is the photosensitizer used. Employing different imidazoacridinone derivatives, we have shown that fast intake and slow clearance from the treated yeast is a crucial parameter. Indeed, the best derivatives, in terms of PDI efficacy, could be detected within the treated cells, even after prolonged incubation (up to 30 min). This observation is in contrast with the previously studied cationic porphyrin TriP[4], which was slowly taken up and inefficiently accumulated by yeast cells. It has been suggested that membrane damage is responsible for the killing of *C*. *albicans*(21). These authors also showed that vacuoles remained intact during PDI treatment. Notably, imidazoacridinone accumulation can be easily reversed as demonstrated after washing the cells with PBS, which decreased the observed imidazoacridinone-dependent phototoxicity. The data obtained here and particularly the lack of inhibition by verapamil, suggested that imidazoacridinones are largely imported into cells via spontaneous diffusion through the membrane. In contrast to hypericin [42], where the photosensitizer localized within a large intracellular patch, most likely within a vacuole, imidazoacridinones localized in other cytosolic organelles with the vacuole remaining unstained. Phthalocyanine Pc4, another frequently used photosensitizer, was shown to localize in the mitochondria[43]. Our most active derivative, imidazoacridinone 1330, showed an intracellular accumulation pattern, most likely in the cytoplasm. This unique pattern of intracellular distribution might be associated with a dramatic increase in lethality of photosensitized cells. The remaining IAs exhibited a rather dispersed pattern of cytoplasmic distributions. A recent study on human ovarian carcinoma cells treated with 3,6-bis(imidazolidine)acridine, which is structurally similar to imidazoacridinones, indicated that this compound primary localizes to lysosomes and mitochondria, but not in the nuclei [44]. Similarly, the lysosomal localization of imidazoacridinones was observed in human A549 cell line [45]. Therefore, it is likely that 1330 localizes in *Candida* lysosomes, and this localization pattern results in highly effective lethal photosensitization (as further discussed below).

The key mechanism of drug resistance in *C*. *albicans* is the low intracellular accumulation of antifungal agents due to elevated levels of drug efflux pumps such as ABC (ATP binding cassette) transporters, primarily of CaCdr1p and CaCdr2p [46]. Following a genetic modification, *Candida albicans* strains overexpressing ABC efflux pumps (CaCDR1/CaCDR2) resisted methylene blue-mediated photodynamic action [47]. Photosensitizers comprising substrates for ABC-type transporters therefore have limited efficacy for antifungal photodynamic treatment [47]. As shown here, imidazoacridinones are not substrates for ABC transporters, as indicated by the lack of verapamil inhibition, which blocks the cellular efflux pump CaCDR1. The observed decrease in imidazoacridinones accumulation in *C*. *albicans* might reflect either the disturbed uptake of these compounds in the presence of verapamil or CaCDR1-independent leakage. The observed differences in accumulation profiles of particular imidazoacridinones in the presence and in the absence of verapamil are relatively small, and might therefore reflect the multi-effect action of verapamil on yeast cells. CaCDR1 transporters have been implicated in multiple functions, including the transport of inorganic ions, efflux of steroids, and phospholipid translocation. These mechanisms might indirectly contribute to the small differences observed in the absence and presence of verapamil [48]. Taken together, CaCDR1 is unlikely to expel the studied imidazoacridinones.

The first step in photodynamic action is light absorption by a photosensitizer. In the presence of oxygen, excited photosensitizers act according to two processes: type I and/or type II. As a result of electron transfer, radical species are generated (type I); however, energy transfer between the excited triplet state of the photosensitizer molecule and the ground state molecular oxygen leads to the formation of singlet molecular oxygen (type II). Both types of reactive oxygen species: oxidizing radicals and singlet oxygen, react with DNA, proteins, lipids or other cellular biomolecules. In various photodynamic phenomena, singlet oxygen is typically considered as the reactive oxygen species responsible for critical cellular damage [49]. The production of singlet oxygen is important in PDI, as there are no known resistance mechanisms for the detoxification of this compound [18]. The quantum yield of singlet oxygen production upon the photoexcitation of imidazoacridinone derivatives was low compared with the known efficient producers of singlet oxygen, such as TMPyP or Rose Bengal photosensitizers (Fig 5). However, even the relatively inefficient production of singlet oxygen could have significant biological impact when this species is generated in proximity to molecular targets that readily undergo oxidative modifications [50–51]. This phenomenon was observed in the case of 1330 and 1330H,which produced similar amounts of singlet oxygen, and showed similar levels of accumulation in yeasts cells, but have different localization pattern. As a result, significant differences in the photokilling efficacy of these compounds were observed (Fig 3 and Table 1). Although all imidazacridinones examined in the study produced ^1^O_2_, the yield of different compounds varied, ranging from 2–16%. There was no obvious correlation between the efficiency of singlet oxygen photogeneration and the photokilling efficacy of imidazoacridinones. However, it should be emphasized that the luminescence signal of ^1^O_2_ might be affected by differences in *in vitro* and *in vivo* models, as shown for porphyrin XF73-based lethal photosensitization process [52]. Additionally, the potential role of free radicals in the photodynamic killing of the yeast should be considered. The efficiency of the imidazoacridinones to photogenerate superoxide anion is even lower than that of singlet oxygen. In the absence of electron donors, such as NADH, the compounds photogenerate superoxide anion with at least ten-fold lower efficiency than riboflavin, which has quantum efficiency for such a process ~ 1% [53]. Although in the presence of NADH, the efficiency of the superoxide anion photoformation increases two orders of magnitude, the resulting quantum yield is only several per cents at most. This said, the efficiency of strongly oxidizing free radicals, such as hydroxyl radical for inducing peroxidation of unsaturated lipids or proteins could be significantly higher than that of singlet oxygen. This is because such a process proceeds via a free radical chain reaction mechanism, in which the initial oxidative damage is propagated via secondary reactions involving carbon-centered radicals and oxygen molecules in their ground state [54]. Hydroxyl radicals are formed during processes, such as the metal ion-catalyzed free radical decomposition of hydrogen peroxide, which is the product of superoxide anion dismutation [54]. The photodynamic mechanism of yeast killing mediated through imidazacridinones might be similar to that of fullerenes, which show relatively low efficiency as photosensitizers operating via type II photochemistry [55]. Indeed, type I photoreactions might be as effective as type II, and possibly even more effective in antimicrobial PDI [56]. For some groups of photosensitizing compounds such as thiazins, xanthenes or acridines, it has been shown that oxygen radicals, primarily superoxide anions, were generated [57]. Accordingly, we detected superoxide anion production through imidazoacridinones. As one of the products of imidazoacridinone photoactivation the superoxide anion can easily dismutate to form more stable hydrogen peroxide in aqueous solutions. Hydrogen peroxide can decompose in a free radical process to create the most reactive oxygen species (ROS), hydroxyl radical. The presence of these ROS might be responsible for the efficacy of imidazaocridinones photokilling.

Here, we have characterized for the first time the mechanism underlying IA action against *C*. *albicans*. We have shown that structurally different imidazoacridinone derivatives are able to inactivate *C*. *albicans* cells in light dependent manner. Moreover, we provide indications that fluence rate should be considered as a crucial parameter to improve efficiency of IA-mediated PDI. A correlation between ROS production by particular IA derivative and cell killing was not observed. Interestingly, we have shown, that IA accumulation and localization events are critical factors for cell killing, which is in accordance with results obtained for other photosensitizing agents. The results presented here show that IA not only efficiently accumulate in cells but importantly they are not actively expelled from *C*. *albicans* cells via CaCDR1 ABC transporter. Therefore, IA are promising candidates for the long-term treatment of localized infections. Overall, this study contributes to a better understanding of the mode of action of imidazoacridinones, and suggests that these compounds are potential reagents for the photokilling of *C*. *albicans*.

## Supporting Information

S1 Table Accumulation and phototoxic effect of imidazoacridinone derivatives in *Candida albicans* ATCC 14053 cells. (DOCX)

S2 Table Accumulation and phototoxic effect of imidazoacridinone derivatives in *Candida albicans* ATCC 90028 cells. (DOCX)
