# Supplementary material for: Photodynamic Inactivation of Candida albicans with Imidazoacridinones: Influence of Irradiance, Photosensitizer Uptake and Reactive Oxygen Species Generation
Source: PLoS One. 2015 Jun 8;10(6):e0129301. doi: 10.1371/journal.pone.0129301 (PMC4459871; doi:10.1371/journal.pone.0129301)
Supplement: S1 Table — (DOCX) [file pone.0129301.s001.docx]

**S 1 Table Accumulation and phototoxic effect of imidazoacridinones in *Candida albicans* ATCC 14053 strain.**

| **IAs** | **Initial IAs conc. 50 µM = 0.05 µmol/10^6^ cells** | | **Initial IAs conc. 100 µM = 0.1 µmol/10^6^ cells** | | **PDI effect (20 J/cm^2^, 7 mW/ cm^2^)** | |
| --- | --- | --- | --- | --- | --- | --- |
|  | **IAs accumulated in cells^a^** | **IAs remained after washing^a^** | **IAs accumulated in cells^a^** | **IAs remained after washing^a^** | **50 µM IA** | **100 µM IA** |
|  | **µmol/10^6^ cells ± SD** | | **µmol/10^6^ cells ± SD** | | **Reduction in survival [log_10_ units CFU/ml]^b,c,d^** | |
| **1330** | 0.032 ±0.002 | 0.022 ±0.003 | 0.062 ±0.003 | 0.045 ±0.001 | >5±0.00 | >5±0.00 |
| **1415** | 0.020 ±0.002 | 0.010 ±0.001 | 0.045 ±0.002 | 0.027 ±0.001 | >5±0.00 | >5±0.00 |
| **1558** | 0.030 ±0.001 | 0.019 ±0.000 | 0.056 ±0.002 | 0.036 ±0.002 | >5±0.00 | >5±0.00 |
| **1330h** | 0.039 ±0.001 | 0.034 ±0.001 | 0.081 ±0.001 | 0.072 ±0.002 | >5±0.00 | >5±0.00 |
| **1415h** | 0.025 ±0.000 | 0.016 ±0.001 | 0.053 ±0.001 | 0.038 ±0.001 | >5±0.00 | >5±0.00 |
| **1558h** | 0.028 ±0.001 | 0.016 ±0.001 | 0.051 ±0.003 | 0.029 ±0.005 | >5±0.00 | >5±0.00 |
| **1610** | 0.026 ±0.002 | 0.011 ±0.000 | 0.049 ±0.003 | 0.028 ±0.004 | >5±0.00 | >5±0.00 |
| **1610h** | 0.029 ±0.003 | 0.015 ±0.001 | 0.051 ±0.003 | 0.031 ±0.003 | >5±0.00 | >5±0.00 |
| **1611** | 0.006 ±0.002 | 0.004 ±0.002 | 0.015 ±0.002 | 0.010 ±0.002 | 0±0.63 | 0±0.26 |

IAs – imidazoacridinone derivatives; PDI – antifungal photodynamic effect, The values were calculated by subtracting log_10_ CFU/ml of tested samples from those of untreated controls (0 J/cm^2^; 0 µM IA). At least three biological replicates were used for calculation of the mean reduction values.

^a^mean values of three replicates ± standard deviation of the mean

^b^in samples incubated in the dark without imidazoacridinone derivatives, the number of *C. albicans* cells was c.a. 5x10^6^ CFU/ml

^c^in the samples incubated in the dark with 50 µM or 100 µM of each imidazoacridinone derivative, the number of cells was c.a. 5x10^6^ CFU/ml

^d^in the samples exposed to light only (20J/cm^2^, 7mW/cm^2^), the number of *C. albicans* cells was c.a. 5x10^6^ CFU/ml
